# Kawasaki Disease Shock Syndrome: A Nine-Year-Old Girl With Atypical Presentation of Kawasaki Disease in Emergency Department

**DOI:** 10.7759/cureus.15471

**Published:** 2021-06-05

**Authors:** Sunil Shah, Divya Karki, Sumit Gami, Himal Panth, Nischal Neupane

**Affiliations:** 1 Medicine, California Institute of Behavioral Neurosciences & Psychology, Fairfield, USA; 2 Medicine, Ministry of Health, Malé, MDV; 3 Pediatrics, Ministry of Health, Malé, MDV; 4 Medicine, Universal College of Medical Sciences, Bhairahawa, NPL; 5 Internal Medicine, Nidan Hospital, Lalitpur, NPL

**Keywords:** kawasaki disease, kawasaki disease shock syndrome, shock, systemic inflammation, vasculitis

## Abstract

Kawasaki disease (KD) is an acute rheumatological illness usually affecting children between six months and five years of age. It is a vasculitis syndrome of medium-sized vessels that has typical clinical characteristics such as fever, rash, cervical lymphadenopathy, conjunctivitis, and mucosal changes. However, sometimes, it may present with the features of shock when it is known as Kawasaki disease shock syndrome (KDSS). The actual etiology of this disease is still unknown. The primary treatment of this disease is aspirin and intravenous immunoglobulin (IVIG). The most common and serious complication of KD is cardiac complications which can be avoided by IVIG if given on time. KDSS is the other rare but serious early complication that can be presented to the ED as an initial presenting feature. Early diagnosis of KDSS in the ED and its treatment is very important to prevent early and late complications, including cardiac complications of this disease. Although the usual age group for this disease is under five years, here we have presented a rare case of KDSS in a nine-year-old female child.

## Introduction

Kawasaki disease (KD), also known as mucocutaneous lymph node syndrome, is an acute rheumatological illness mostly affecting children [1]. It usually presents in children between six months and five years of age, but less commonly, it can affect children of ages beyond this range and rarely adults [2]. It is characterized by acute onset of fever with distinctive clinical characteristics such as fever, rash, cervical lymphadenopathy, mucosal changes, and conjunctivitis. The presence of these features helps to diagnose this disease. However, certain atypical features are not included in the classical criteria, which can be present in a small subset of children with KD. One such feature is hemodynamic instability which is also known as KD shock syndrome (KDSS) that was first described and reported by Kanegaye et al. in 2009 [3]. Cardiac diseases are the most common and serious complication of KD; however, we should also consider KDSS as an important complication that sometimes presents as a shock in the ED. Here, we present a case of a relatively rare complication of a rare disease as an initial presenting feature, KDSS, in a nine-year-old female child.

## Case presentation

A nine-year-old female child from eastern Nepal was brought to our hospital with complaints of fever for seven days. She was apparently well one week back, after which she developed a fever which was, according to her parents, high grade and almost continuous with only mild control of fever with over-the-counter fever medicines. However, the temperature was not documented at home.

On day three of illness, she developed generalized red patches and papules all over her body, including her palm and soles. She was taken to a local hospital for treatment with these complaints. She got admitted and was treated with antibiotics. The antibiotics and antipyretics given in the local hospital could not adequately control her fever, and her condition progressively deteriorated. Therefore, after two days of admission to the local hospital, she was referred to our center.

She was presented to our hospital in the ED, where she was found to have features of shock such as prolonged capillary refill time (CRT), hypotension, and tachycardia. The blood pressure, temperature, oxygen saturation, and pulse rate during the initial assessment of the patient at the ED were 80/50 mmHg, 38.5-degree centigrade, 98%, and 110 beats per minute, respectively. A single bolus of fluid (20 ml/kg) was given quickly, then blood investigations were sent, and the child was shifted to Pediatric Intensive Care Unit (PICU) for monitoring. The significant blood test findings are as shown in Table 1.

**Table 1:** Blood test results at presentation and third day of admission. ALT: Alanine aminotransferase; AST: Aspartate aminotransferase; CRP: C-reactive protein; ESR: Erythrocyte sedimentation rate; gm/dl: Grams per deciliter; Hb: Hemoglobin; Mcl: microliter; WBC: White blood cells.

Blood investigations	At presentation	Third day
Hb	9.5 gm/dl	8.9 gm/dl
Total WBC count	12500/mcl	18650/mcl
Neutrophil	80%	86%
Lymphocyte	10%	10%
Platelets	140 x 10 (3)/mcl	202 x 10 (3)/mcl
CRP	Positive	-
ESR	120 mm at the first hour	-
ALT	32 units per liter	-
AST	34 units per liter	-

On further examination after stabilization, she was found to have bilateral cervical lymphadenopathies, cracked lips, red strawberry-like tongue, and perianal excoriation. She also had hepatomegaly along with puffiness of the face and edematous limbs. Echocardiography was normal. Since the inflammatory markers were very high and features of KD were present, the child was given intravenous immunoglobulin (IVIG) at a dose of 2 gm/kg with KDSS as a diagnosis. Aspirin (100 mg/kg/day) was also started, and fever was monitored. After 24 hours of IVIG, the fever subsided.

Since the child presented with atypical features of KD (hypotension and shock), streptococcal toxic shock syndrome was also considered. Thus, she was prescribed crystalline penicillin also. She was discharged after four days of admission as she did not have a fever, vital signs were stable, and general well-being was improved. She was asked for follow-up after two weeks and six weeks to monitor any cardiac complications due to KD; however, echocardiography was absolutely normal on follow-up visits. Aspirin was stopped after six weeks as the echocardiography finding was found to be normal.

## Discussion

KD was first described in Japan by Dr. Tomisaku Kawasaki [4]. KD is a systemic vasculitis of a medium-sized vessel and has an unknown etiology [4]. It has a higher incidence in children from Asia compared to the Western world. The incidence rates of KD per 100,000 children under five years of age are highest in children of Japanese ancestry [5]. Japanese KD nationwide survey reported an increased incidence of KD over time from 2008 to 2015. The incidence rate of KD in 2008 was 218.6 per 100,000; however, the rate progressively increased to 243.1 and 330 in 2011 and 2015, respectively [5,6]. The incidence appears to have remained almost the same with time in the United States. In 2003 and 2012, the KD-associated hospitalization rate in the United States for children less than five years of age was 19.7 and 18.1 per 100,000 children, respectively [7]. In Nepal, the true burden of Kawasaki disease is unknown since many cases go undiagnosed and untreated due to a lack of knowledge regarding this entity [8].

Although many theories such as vaccine exposure theory, infectious theory, and immune factor dysregulation theory have been postulated, the exact cause of this disease is unknown [7]. However, the theory remains that an unknown stimulus triggers an inflammatory cascade resulting in activation of the innate and adaptive immune system, ultimately leading to activation of several inflammatory cytokines affecting the systemic arterial vascular wall [2]. Furthermore, the exact etiology of KDSS is also unknown, but there are hypotheses supporting vasculitis with capillary leakage, myocardial function defect, and cytokine storm [3].

The typical clinical features of KD include fever of unknown etiology, which ranges from 38°C to 40°C and persists for five days or more. In general, the patient has remittent or continuous fever usually with no prodromal symptoms such as cough, sneezing, or rhinorrhea. Other important clinical features and diagnostic criteria of KD are as shown in Table 2 [9].

**Table 2:** Clinical features and diagnostic criteria of Kawasaki disease.

Diagnostic criteria	Clinical characteristics
Polymorphous rash	Can present in many forms, such as urticarial exanthema with large erythematous plaques and morbilliform maculopapular rash. In rare cases, it may present as erythema multiforme-like rash with central clearing or iris lesion
Bilateral hyperemia of conjunctiva	Bilateral conjunctival injection within 2-4 days of onset, not associated with exudate, each capillary vessel clear because of individual capillary dilatation, limbic sparing
Mucosal changes	Redness, dryness, fissuring, and bleeding of the lips, diffuse erythema of the oropharyngeal mucosa, strawberry-like tongue
Changes in peripheral extremities	Diffuse erythema of the palms and soles and/or indurative edema of the hands and feet
Cervical lymphadenopathy	Non-suppurative (≥1.5 centimeters), typically unilateral

The patient is diagnosed with KD when they have ≥ five days of fever and ≥ 4 of the five principal clinical features shown in Table 2 [9]. In some cases, experienced pediatricians diagnose KD before the fifth febrile day when other principal symptoms and laboratory data compatible with KD are also present. Other significant multisystemic findings like heart murmur, cardiomegaly, electrocardiography changes, cough, diarrhea, joint pain, and neurological findings should be considered during the evaluation of the patients.

Leukocytosis with increased neutrophils and decreased lymphocytes along with increased erythrocyte sedimentation rate and positive C-reactive peptide are common findings, as shown in our case. Hypoalbuminemia, increased α2-globulin, a slight decrease in erythrocyte and hemoglobin levels are the lab finding expected in KD. There can also be proteinuria, increased leukocytes in urine sediment, increased serum transaminases, and decreased hemoglobin [9].

Although the most common and serious complication of KD is cardiac complications, including coronary artery aneurysm, myocarditis, myocardial infarction, and sudden cardiac death, KD can also be presented as KDSS [10]. KDSS has a clinical presentation of KD with features of shock such as hypotension, increased CRT, and increased pulse rate. The primary treatment of KD includes IVIG and aspirin, along with management of shock in the case of KDSS. IVIG is most effective when administered within 10 days of onset of fever, and its use decreases the risk of coronary artery aneurysm formation from 20-25% to 3-5% [7]. In addition to IVIG and aspirin, adjuvant therapy, when not responsive to primary treatment, includes corticosteroids, TNF inhibitor, interleukin-1 inhibitor, and calcineurin inhibitors. Aspirin at a low dose is continued to prevent the risk of thrombosis until no adverse cardiac outcome is found on the final echocardiography at follow-up after 6-8 weeks [7].

The extent and severity of coronary artery involvement at diagnosis and follow-up determine the prognosis of children diagnosed with this disease [8]. The case-fatality rate is less than 0.2% in the United States and Japan, while it is largely unknown in many developing countries like Nepal [8,11]. The principal cause of death in KD is cardiac complications due to coronary artery occlusion, while the shock that can be associated with KD by itself may lead to cardiorespiratory failure and death.

## Conclusions

The atypical presentation of KD as KDSS, similar to our case, can be an initial presenting feature in KD. Although usually, KD occurs in children aged between six months and five years, children of age greater than five years can be affected by KD. The early recognition of KDSS and appropriate shock management in the ED followed by treatment with IVIG and aspirin is very important to prevent adverse clinical outcomes and long-term cardiac complications.
